# Do NICU developmental care improve cognitive and motor outcomes for preterm infants? A systematic review and meta-analysis

**DOI:** 10.1186/s12887-020-1953-1

**Published:** 2020-02-13

**Authors:** Farin Soleimani, Nadia Azari, Hesam Ghiasvand, Amin Shahrokhi, Nahid Rahmani, Shiva Fatollahierad

**Affiliations:** 1 Pediatric Neurorehabilitation Research Center, University of Social Welfare and Rehabilitation Sciences, Tehran, Iran; 2 Health Economics Group, Institute of Health Research, Medical School, Saint Luke’s Campus, University of Exeter, Exeter, UK; 3 Department of Physiotherapy, Pediatric Neurorehabilitation Research Center, University of Social Welfare and Rehabilitation Sciences, Tehran, Iran

**Keywords:** Preterm infants, Developmental care, NICU, Interventions, Bayley scales of infant development, Meta-analysis, Systematic review

## Abstract

**Background:**

The aim of this study was to review the effects of developmental care in neonatal intensive care unit (NICU) setting on mental and motor development of preterm infants.

**Method:**

We searched PubMed, EMBASE, CINAHL, Scopus, Web of Science and Cochrane library until October 8th 2017, and included randomized controlled trials that assessed effects of developmental care in NICU on mental and motor development of preterm infants at 12 and 24 months of age, using the Bayley scale of infant development in this systematic review. In addition, data were pooled by random effects model and Standardized Mean Difference (SMD) with 95% confidence intervals (CI), calculated for meta-analysis.

**Results:**

Twenty one studies were eligible to be included in this systematic review; however, only thirteen studies had data suitable for meta-analysis. According to statistical analysis, developmental care in NICU improved mental developmental index (MDI) (standardized mean difference [SMD] 0.55, 95% confidence interval [CI] 0.23–0.87; *p* < 0.05), and psychomotor developmental index (PDI) (SMD 0.33, [CI] 95% CI 0.08–0.57; *p* < 0.05) of BSID at 12 months of age and PDI at 24 months of age (SMD 0.15, 95% CI -0.02–0.32; *p* < 0.1) of preterm infants. However, the benefit was not detected at 24 months of age on MDI (SMD 0.15, 95% CI -0.05–0.35; *p* = 0.15).

**Conclusion:**

Current evidence suggests that developmental care in only NICU setting could have significant effect on mental and motor development of preterm infants, especially at 12 months of age. However, because of clinical heterogeneity, more studies are needed to evaluate the effects of developmental NICU care in the development of preterm infants.

## Background

Every year, an estimated 15 million infants are born preterm [1]. The highest rate of preterm births is in sub-Saharan Africa and South Asia (over 60%) [1]. The preterm birth rate in developing countries varies widely and has a different pattern than developed countries [2].

In recent years, the mortality rate in preterm infants were reduced by advanced perinatal care, but the developmental morbidity is remarkably high [3, 4]. In preterm infants, in addition to cerebral palsy, hearing loss, visual impairment, and growth retardation, long-term follow-up studies have identified important developmental disorders [5]. A recent study showed that more than 25% of neonates born between 28 and 32 weeks of gestation have developmental disorders at the age of 2 years old, and this ratio reaches 40% at the age of 10 [6].

Each sensory experience, proportionate or disproportionate, in the infant’s brain causes a behavioral response, which itself results in another sensory experience. When the preterm infant has sensory experiences that are disproportionate to its developmental stage, its neurodevelopment will be different from when it is in the protective environment of the uterus. Therefore, it is not surprising to see different neurodevelopmental outcomes in preterm infants compared with term ones [7]. In addition, fetal nervous system is in a very active stage of development during the third trimester. Therefore, the nervous system is vulnerable due to the immature and rapid growth in preterm infants [8].

Neurological care includes strategies that can prevent the neuronal death [9]. These strategies are interventions that protect the evolving brain or help the brain to reduce the death of neurons after trauma and improve their performance by creating new communication pathways. The more immature is the infant, the more vulnerable is its brain, and the more necessary is neurological care for it [10]. In order to prevent these complications, various methods have been proposed and implemented by researchers over the past few decades. Most of these methods are developmental interventions or cares in infants admitted to the NICU (Neonatal Intensive Care Unit). Developmental cares are methods that are intended to adjust the NICU environment to diminish the stress, support the behavioral organization, improve physiological stability, keep sleep rhythms, and promote neural growth and maturation of infant [7, 11]. In this type of care, the training and participation of parents or caregivers are critical for the social, emotional and physical health of the infant, and are important factors in the family-based care process [12]. The goals of developmental care for the family are to encourage and support parents in the primary caregiver role, and enhance family emotional and societal well-being [11, 13].

It appears that intense sensory impacts of NICU have devastating effects on neurodevelopmental outcomes in preterm infants. However, according to research, it is unclear which of the severe [8] or mild stimuli or inappropriate stimuli [14] can be most harmful to neonatal development.

The effectiveness of developmental care in preterm infants has been investigated in a number of previous systematic reviews [13, 15–21]. Symington et al. investigated the effects of five core measures on preterm infants during NICU admission, emphasizing the short-term medical and neurobehavioral development outcomes. Symington et al. concluded that there is limited evidence to support the benefits of developmental care in improving the cognitive, motor and behavioral development in infants at the time of discharge, but negative effects have not been reported in this regard and improved neurodevelopmental outcomes to 24 months corrected age [13, 21].

Lavallée et al. examined the Developmental Care Interventions (DCI) outcomes by presenting a comprehensive narrative review of recent findings on the effectiveness of DCI on stress relief, sleep promotion, and neurodevelopmental outcomes. The main limitation of this study was the lack of a systematic method of review. Therefore, the quality of the papers examined has not been evaluated with clear criteria or standard tools and there might be bias in their report [17].

Orton, Spittle, and their colleagues investigated the effects of interventions after discharge on the cognitive and motor development of premature infants in three age groups; infancy (0 to < 3 years old), pre-school (3–5 years old), and school (5–13 and 13–18 years old) and concluded that early developmental interventions improved cognitive outcomes at infant age, and at pre-school age. However, the benefit was not sustained at school age. In addition, developmental interventions had little effect on motor outcome at infancy or school age [15, 16].

Jacobs et al. studied the long-term developmental effects during school and short-term medical and developmental effects of NIDCAP in comparison with routine care in preterm and low-birth-weight infants. The study concluded that there is insufficient evidence to support NIDCAP in improving short-term medical results and neurodevelopmental enhancement of preterm infants at school [18].

In the case of family-centered interventions, Vanderveen et al. have recently conducted a systematic review of early intervention programs in preterm infants focusing on training the parents. The review identified studies that used various interventions, including training the parents, infant’s stimulation by parents, home visits, and individual developmental care. The meta-analysis of the studies showed that early interventions had improved cognitive and motor performance of preterm infants at 12 and 24 months of age, but their effects are not sustainable until the school age [19].

Among these reviews, only Symington et al. examined effects of developmental interventions during NICU setting; however, they only searched for interventions such as control of external stimuli (vestibular, auditory, visual, tactile), clustering of nursery care activities, positioning or swaddling of the preterm infant and individual strategies such as the ‘Newborn Individualized Developmental Care and Assessment Program’ (NIDCAP), and did not use an extensive search method [13, 21].

The results of the two reviews by Symington et al. indicate that there is very limited evidence that NIDCAP have positive long-term effects on the behavior and movement of preterm children at 5 years corrected age, but there is no effect on their cognition at 5 years corrected age. Also, other individualized developmental care interventions have demonstrated some effects in enhancing neuro-developmental outcome [13].

In addition, in other systematic reviews, the developmental outcomes of post-NICU discharge interventions have been addressed [15–20].

Various assessment tools are used to assess the effectiveness of developmental outcomes in infants and children. These tools have different accuracy and validity, and there is concept that these tools may not have enough sensitivity to identify or monitor the improvement of minor problems as long-term neuro-developmental outcomes [15, 22].

The Bayley Scales of Infant Development (BSID) [23–25] is the best measure for the assessment of infants and the most widely used measure to assess developmental improvement. BSID is frequently viewed as the end point of follow-up in high-risk infants [26]. The BSID-I/II includes two scales, including the Mental Developmental Index (MDI) and Psychomotor Developmental Index (PDI), The Bayley-III comprises three scales, including a cognitive, language, and motor Composite.

When an intervention begins for infants at risk of developmental disorders, the intervention has a preventive focus and has strategies to minimize developmental complications. Thus, it is important for the care provider to assess the effectiveness of these programs in at risk infants. Before being able to support this developmental care as one of the care goals in patients we need evidence to show that the developmental care has proper effects on short- and long-term developmental outcomes. However, the effectiveness of these developmental care programs in preterm infants in the NICU has not been fully approved.

Considering the importance of developmental care on reduction of neuro-developmental disorders in preterm infants, and various and more precise clinical trials conducted in this field in recent years, we decided to investigate the effect of developmental care in NICU setting on first 2 years of mental and motor development of preterm children by a systematic review, with an analysis of the risk of bias and an extensive search method.

In this review, developmental care was considered as a program that begins in the NICU with the aim of support and promoting development, and we analyzed papers that tracked the intervention effects of various types of developmental care on the neonatal development, examining them with different editions of the BSID.

## Methods

### Search strategy and selection criteria

We searched PubMed, EMBASE (through OVID), CINAHL (through EBSCO), Scopus, Web of Science and Cochrane library up to October 8th, 2017 for relevant articles. The search strategy consisted of text words, such as premature, preterm, low birth weight and Bayley; and relevant medical subject headings (MESH). The complete search strategy is shown in Additional file 1: Table S1. The reference lists of included studies were searched in person for relevant articles. There was no language restriction in search. However, we only screened articles that their abstracts were in English.

Randomized controlled trials (RCTs) that assessed the effects of developmental care in NICU setting on development of preterm neonates (< 37 weeks) were included in this systematic review. The developmental care were consisted of environmental stress controls; individualized approaches, such as NIDCAP; integration of parents, such as mother training for understanding behavioral cues of their infants; and behavioral techniques on neonates [11]. The BSID was the developmental assessment tool used in included studies. Studies that included neonates with major brain abnormalities or any other health situation influencing neurodevelopment, such as intra-ventricular hemorrhage greater than II, broncho-pulmonary dysplasia (BPD) or brain malformation were excluded. Furthermore, studies that used developmental assessment tools other than BSID or published before 1970 were also excluded. The primary outcomes of interest in this systematic review were mental and motor development of preterm infants that were assessed by BSID. The protocol of this review will be published in Iranian Journal of Child Neurology.

### Data extraction

This study employed Preferred Reporting Items for Systematic Reviews and Meta-Analyses (PRISMA) [27] to identify relevant articles and report the screening process. The records of search were exported to Endnote and were screened by two reviewers. The eligible studies were read in full and relevant studies were included for assessment in this systematic review. Any disagreement about selecting an article was resolved through discussion. The data of included studies were extracted in pre-designed forms by two authors. These data included the author, publication year, intervention date, country, design, sample size, intervention type, intervention duration, intervention intensity, assessment tool, assessment times and results. Any discrepancies between the extracted data were discussed to reach a consensus.

The risk of bias of included studies was evaluated by “Cochrane collaboration risk of bias tool” [28, 29]. The domains of risk of bias tool are “random sequence generation,” “allocation concealment,” “blinding of participants and personnel,” “blinding of outcome assessment,” “incomplete outcome data” and “selective reporting.” The risk of bias was classified as “low,” “unclear” and “high” risk in each domain for an outcome in the included studies. Review Manager 5.3 was used to illustrate the risk of bias graphs. Risks of bias of studies were evaluated by two authors and disagreements were resolved through discussion.

### Data synthesis and statistical analysis

Qualitative Synthesis was done using data from all included studies to draws the findings from individual studies together however quantitative analyses included studies that had numerical data. The meta-analysis as quantitative analyses was performed with studies that their data were documented or were obtained through contact with the author. We performed a random effects model meta-analysis for estimating the Standardized Mean Differences (SMD) in STATA.

Subgroup analyses were based on risk of bias level, gestational age (≤ and > than 28 weeks of gestational age), birth weight (≤ and > than 1250 g), method of intervention delivery (nurse, mother/nurse, environmental), intervention type (NIDCAP, environmental and others, such as massage therapy, handling and mother training), intervention date (before and after year 2000) and assessment time (12 or 24 months of age). These subgroup analyses were used to find both clinical and statistical heterogeneity between studies.

Assessment times (12 or 24 months of age) were considered to find the long-term effect of developmental care in NICU. If a study didn’t assess the development at 12 or 24 months of age, their data were used in the nearest time point to 12 or 24 months of age. Studies that evaluated the development in less than 6 months of age were excluded from the meta-analysis, because of clinical heterogeneity. Meta-analysis was limited in this review due to the limited number of randomized trials that were included in each subgroup.

In addition, for the interpretation of meta-analysis results, SMDs: 0.2, 0.5 and 0.8; were considered as small, medium, and large intervention effect size respectively [30]. The Publication bias was evaluated with funnel plots and checked with Egger’s test.

### Quality of the evidence

We used Grading quality of evidence and strength of recommendations (GRADE) criteria to assess the quality of evidence [31]. The RCTs are high quality evidence. However, we downgraded the outcomes by one level for serious concerns about inconsistency, indirectness, imprecision, and publication bias criteria. The quality of each outcome is described as high, moderate, low, and very low based on GRADE criteria. We only used low risk studies for the quality assessments.

The risk of bias of each outcome in a study was deduced by defining three main domains in risk of bias tool. These domains were “random sequence generation,” “allocation concealment,” and “blinding of outcome assessment” that were key domains for our systematic review. If all three of these domains were low risk in a trial, the outcome of interest in that trial was considered low risk. If one domain was unclear or high risk, the outcome of interest in that study was considered unclear or high risk respectively.

For determining the heterogeneity, the I^2^ was assessed. If I^2^ was more than 75%, the quality of evidence was downgraded by one level.

## Results

### Study selection

**Fig. 1:**
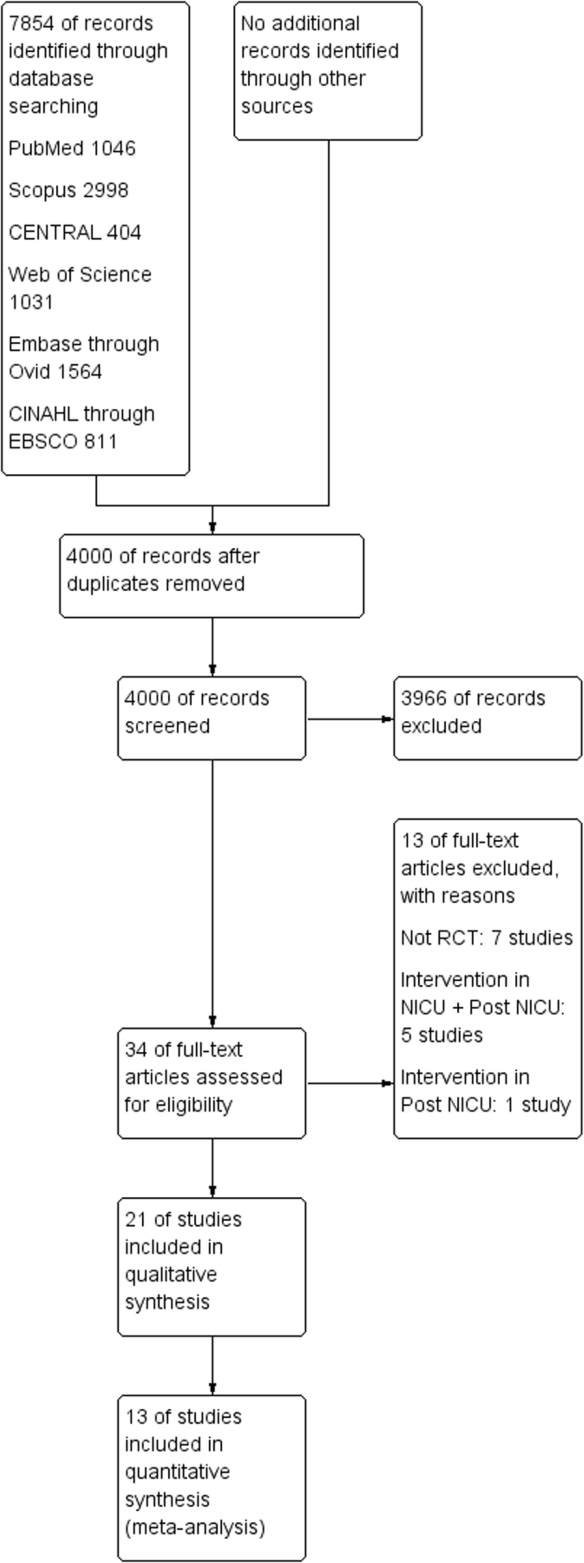
Flow diagram of the study

In this review, 7854 records were identified via the electronic search, after duplication screening, 4000 records remained. Based on the inclusion criteria, 3966 studies were excluded by reading titles and abstracts and 34 articles assessed. Full-texts of 34 articles were read and 13 articles were excluded because of not having RCT design [32–38], intervention in both NICU and post-NICU settings [39–43], and intervention only in post-NICU setting [44]. Finally, 21 studies with 1528 participants were included in this systematic review. The PRISMA flow diagram is presented in Fig. 1.

### Study characteristics

The study characteristics are presented in Table 1. Fifteen studies were in the USA, two in Canada, one in Switzerland, two in Netherlands and one in Brazil. All articles were in English.

**Table 1:** Characteristics of included studies

Author, Year	Country	Inclusion Criteria	Gestational Age at birth(w) (Mean (SD))	Birth Weight(g) (Mean (SD))	Number of participants (Intervention, Control) (n)	Intervention Method	Intervention Delivery	Intervention Intensity	Developmental results
Abou Turk, 2009 [45]	USA	BW: 401–1500 g	C: 26.5 (1.7)I: 25.7 (1.7)	C: 924 (216)I: 806 (236)	34 (18,16)	Noise reduction	Nurse	Continuous	MDI: better in intervention PDI: ND
Als, 1994 [46]	USA	GA: 24–30 wBW: < 1250 g	C: 26.5 (1.4)I: 27.1 (1.6)	NS	38 (20,18)	NIDCAP	Nurse/Mother	Continuous	MDI: better in intervention PDI: better in intervention
Als, 2004 [47]	USA	GA: (28w + 4 d) – (33w + 3 d)	C: 31.83 (1.47)I: 31.22 (1.39)	C: 1730 (350)I: 1648 (232)	33 (18,15)	NIDCAP	Nurse/Mother	Continuous	MDI: better in intervention PDI: better in intervention
Als, 2011 [48]	USA	Severe IUGRGA: 28–33 w	C: 31.69 (2.39)I: 32.06 (2.28)	C: 1122 (336)I: 1083 (343)	30 (12,18)	NIDCAP	Nurse/Mother	Continuous	MDI: better in intervention PDI: ND
Als,2012 [49]	USA	Severe IUGR GA: (26w + 4d) - (33w + 3d)	C: 30.40 (1.91)I: 30.76 (2.78)	C: 1048 (250)I: 997 (324)	30 (13,17)	NIDCAP	Nurse/Mother	Continuous	MDI: better in intervention PDI: ND
Ariagno, 1997 [50]	USA	GA: ≤ 30 w BW: ≤1250g	C: 26.1 (1.8) I: 26.4 (2.1)	C: 811.7 (210.8) I: 884.1 (182.0)	35 (17,18)	NIDCAP	Nurse/Mother	Continuous	MDI: ND PDI: ND
Brandon, 2017 [51]	USA	GA: ≤28 w	C: 26.3 (1.5) I: 26.3 (1.4)	C: 872.7 (232.7) I: 874.1 (219.7)	121 (63,58)	Cycled light ^e^	Nurse	Cycled light ^k^	MDI: NDP DI: ND
Brown, 1980 [52]	USA	GA: ≤ 37 BW: 1000–1750 ^a^	C:31.1 (2.9)I: 31.8 (2.4)	C: 1506 (216)I: 1564 (217)	NS	Infant stimulation, Mother training	Nurse	-5 days/week- twice daily -30 min/session	MDI: ND PDI: ND
Fajardo, 1992 [53] [54]	USA	GA: < 31 BW: < 1300 g + AGA	C: 29.75 (28–31)I: 28.75 (26–30)	C: 1108 (680–1290)I: 1052 (817–1300)	24 (12,12)	Reduce patterned stimuli ^f^	Nurse	4 weeks from 32 to 36 weeks PCA	MDI: ND PDI: better in good state organizer
Feeley, 2012 [55]	Canada	BW: < 1500 g	C: 27.9 (2.2) I: 28.0 (2.3)	C: 979.5 (221.6) I: 982.2 (284.1)	122 (61,61)	Cues and Care trial	Mother	1–2 sessions/ week l	Bayley III: Cognitive: ND Motor: ND Language: ND
Guyer, 2012 [56]	Switzerland	GA: ≤32 0/7	C: 29.5 (2.1)I: 30.6 (0.95)	C: 1284 (346) I: 1439 (299)	37 (17,20)	Cycled lighting	Nurse	Cycled light ^m^	MDI: NDPDI: ND
Kramer, 1975 [57]	USA	GA: < 38 BW: < 1800	C: 33 I: 33	C: 1418 I: 1441	14 (8,6)	Touch, in the form of extra tactile stimulation	Nurse	- Daily for at least 2 weeks − 48 min/session	At transfer to crib MDI: better in interventionPDI: ND At 6 weeks and 3 months: MDI: NDPDI: ND
Maguire Arch Dis Child Fetal Neonatal Ed, 2009 [58]	Netherlands	GA: < 32	C: 29.1 (1.9)I: 29.5 (1.6)	C: 1238.5 (337.2)I: 1248.4 (338.1)	192 (98,94)	Basic developmental care ^g^	Healthcare professionals	Continuous	At 12 monthsMDI: ND PDI: better in intervention At 24 months MDI: ND PDI: ND
Maguire PEDIATRICS, 2009 [59]	Netherlands	GA: < 32	C: 29.3 (1.6)I: 29.6 (1.5)	C: 1247 (340)I: 1263 (311)	168 (84,84)	NIDCAP	Nurse/Mother	Continuous	MDI: ND PDI: ND
McAnulty, 2009 [60]	USA	GA: < 29 BW: < 1250 ^b^	C: 26.20 (1.42) I: 30.3 (6.2)	C: 837 (135) I: 850 (157)	107 (56,51)	NIDCAP	Nurse/Mother	Continuous	MDI: better in intervention PDI: better in intervention
Parker, 1992 [61]	USA	GA: ≤ 36	C: 33.4 (2.4) I: 30.3 (6.2)	C: 1687 (356) I: 1368 (410)	41 (56,51)	h	infant-development specialist	-4 times (2–6 sessions)/ week	At 4 months MDI: better in intervention PDI: better in intervention At 8 months MDI: better in intervention PDI: ND
Peters, 2009 [62]	Canada	GA: ≤ 32 BW: 500–1250	C: 27.0 (2.3)I: 27.5 (1.4)	C: 927.1 (204.0) I: 988.2 (183.7)	120 (60,60)	NIDCAP	Nurse/Mother	Continuous	MDI: ND PDI: ND
Powell, 1974 [63]	USA	BW: 1000–2000 g ^c^	NS	d	NS	Handling	Healthcare professionals/Mother	n	At 2 month MDI: NDPDI: ND At 4 months MDI: better in intervention PDI: better in intervention At 6 months MDI: ND PDI: ND
Procianoy, 2010 [64]	Brazil	GA: ≤32 BW: 750–1500	C: 29.7 (1.62)I: 30.0 (1.55)	C: 1151 (198)I: 1192 (189)	104 (52,52)	Massage therapy	Mother observation	-Four times daily-15 min/session	MDI: better in intervention PDI: ND
Szajnberg, 1987 [65]	USA	AGA GA: 28–32 w	C: 24.3 I: 25.7	NS	25 (12,13)	mother-infant observation ^i^	Mother observation	-Only one session 30–40 min intervention at 34 weeks of age	MDI: ND PDI: ND
Welch, 2015 [66]	USA	GA: 26–34 w	C: 30.7 (2.6) I: 30.8 (2.1)	C: 1474 (439) I: 1426 (3960	150 (78, 72)	FNI ^j^	trained NICU nurses	o	Bayley III: cognitive: better in intervention if score above 85 language: better in intervention if score above 85 motor: ND

^a^Black mother. ^b^ Mechanical ventilation within the first 3 h and for > 24 h in the first 48 h. ^c^ Black infants . ^d^ The stimulated Ss had a slightly higher average birth weight (1769 g as opposed to 1674 g), and a greater proportion of males (53% males as opposed to 37% male). ^e^ start at 28 versus 36 weeks PMA. ^f^ Introduce a defined day- night cycle, Emphasize state contingent nursing care. ^g^ incubator covers and positioning aids. ^h^ Enhance a mother’s ability to provide a more stimulating and nurturing environment . ^I^ mothers observation of Brazelton Neonatal Behavioral Assessment Scale that performed on their own infants. ^j^ facilitating an emotional connection between mother and premature infants. ^k^ 11-h-on, 11-h-off pattern. ^l^ 5 sessions in NICU + 1 session in Post NICU, 45 to 75 min/session. ^m^ 12-h-on, 12-houroff pattern. ^n^ Twice daily (72 h to regain the birth weight), Once daily (regain the birth weight to discharge), 20 min/ session . ^o^ Nurture Specialists facilitated FNI during mother after delivery (mean of 7 days). Nurture Specialists met with FNI mothers an average of 6.4 h/week to facilitate calming sessions

*C* Control, *I* Intervention, *GA* Gestational age, *BW* Body weight, *W* Week, *G* Gram; *IUGR* Intrauterine Growth Restriction *AGA* Adequate for gestational age, *NS* Not specified, Bayley II, *MDI* (Mental Developmental Index), PDI (psychomotor developmental index) subscales, Bayley III Cognitive, language, motor subscales, *PCA* Post Conceptional Age, *NIDCAP* Newborn Individualized Developmental Care and Assessment Program *FNI*, Family nurture intervention; *ND*, No difference; *NICU* Neonatal Intensive Care Unit

With regards to the review inclusion criteria, all included studies used BSID (I, II or III) as developmental assessment tool. Five studies used BSID-I [46, 52, 53, 57, 63], thirteen studies used BSID-II [45, 47–49, 51, 56, 58–62, 64, 65], and two studies used BSID-III [55, 66]. One study used both I and II versions for 12 and 24 months assessment respectively [50]. All studies delivered the intervention in NICU setting. However, in just one study, one session was in post-NICU setting [55]. NIDCAP were used in eight trials [46–50, 59, 60, 62]. Three studies used environmental developmental care, such as noise reduction and cycled lighting [45, 51, 56]. Three studies only trained mothers to understand the behavioral cues of their infants [55, 61, 65] and the rest of studies used other developmental care, such as handling and massage [52, 54, 57, 58, 63, 64, 66].

### Risk of Bias assessment

**Fig. 2:**
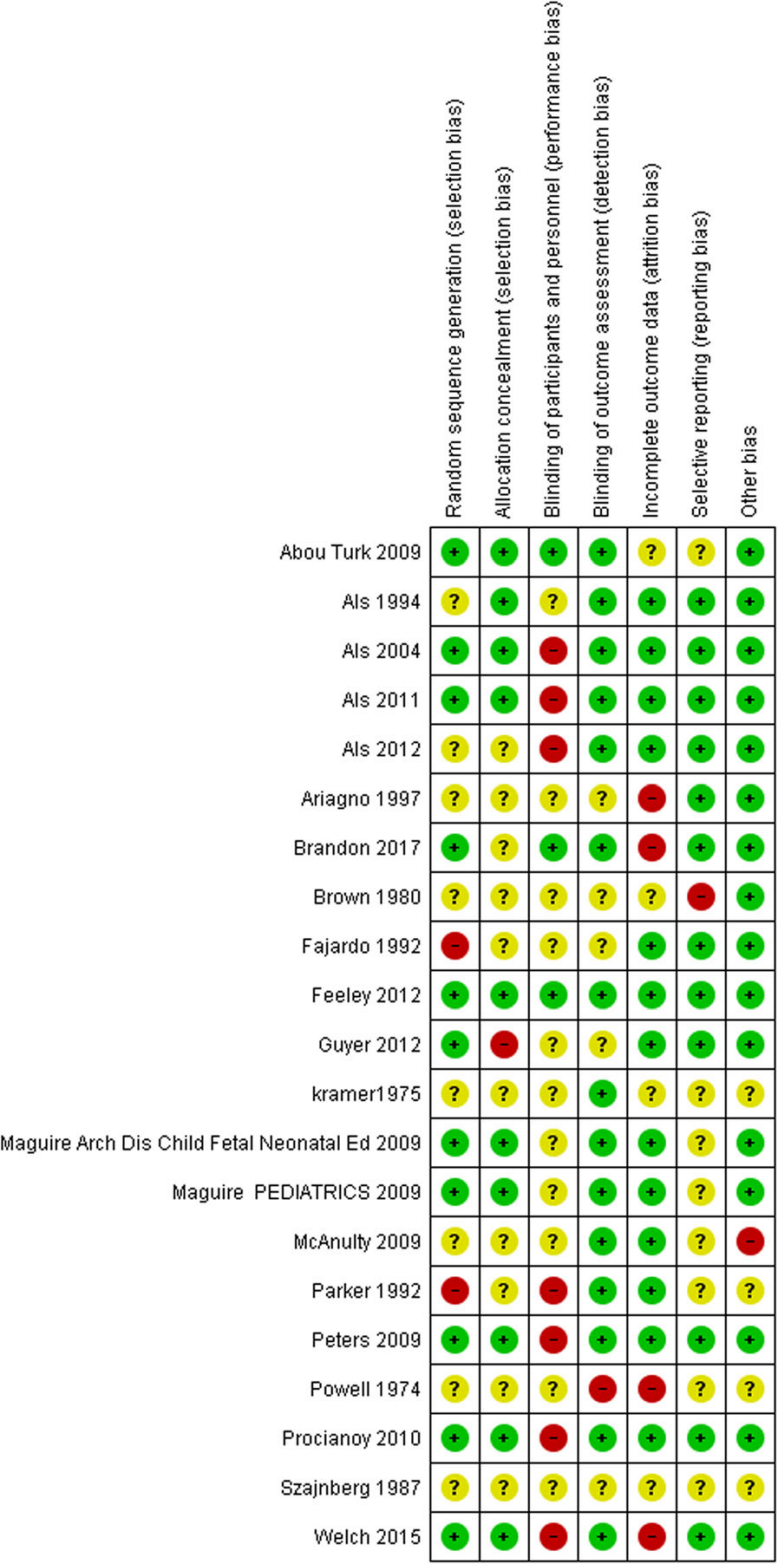
Summary of risk of bias of included studies

**Fig. 3:**
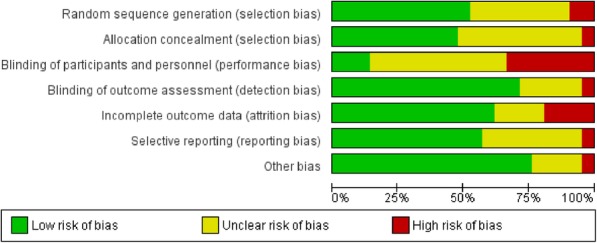
Risk of bias of included studies graph

The risks of bias of included studies were assessed with “Cochrane collaboration risk of bias tool” [28]. The risks of bias of each domain in all studies are summarized in Figs. 2 and 3. With regard to the overall risk of bias of an outcome in a study, nine studies had low risk of bias [45, 47, 48, 55, 58, 59, 62, 64, 66], eight studies had unclear risk of bias [46, 49–52, 57, 60, 65], and four studies had high risk of bias [53, 56, 61, 63].

### Meta-analysis findings

We performed meta-analysis on 13 included studies [46–51, 56, 58–62, 64]. The considered time points for analyzing the results were 12 and 24 months. Developmental assessments of four studies were before 6 months old; because of clinical heterogeneity, their data were not entered in meta-analysis [55, 57, 63, 65]. Three studies’ data were not available or were not reported by means and SD to pool with other studies [45, 52, 54]. In addition, one study used Bayley III for developmental assessment and because of Bayley III uses different scales from BSID I and II, this study’s data were also not pooled with other studies [66].

**Fig. 4:**
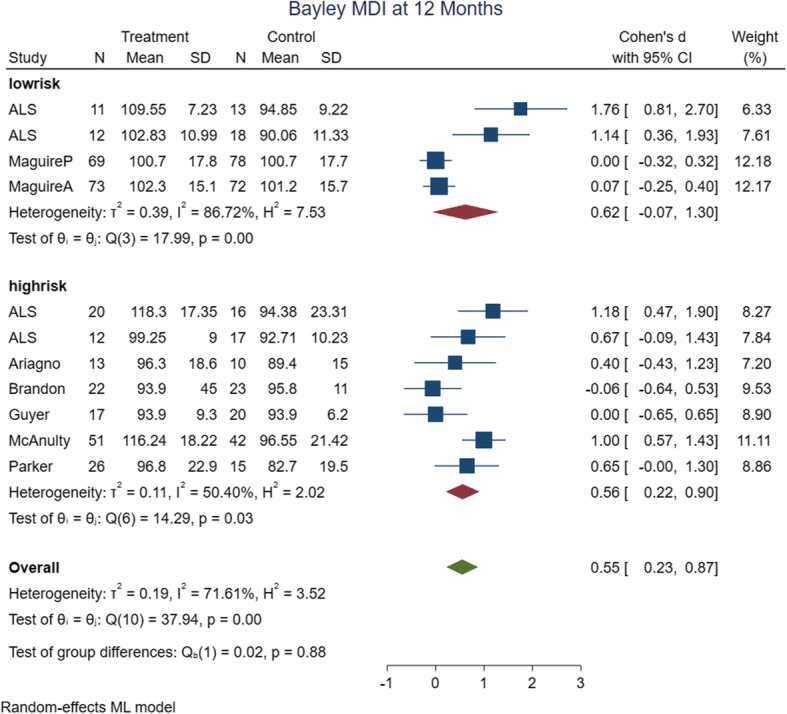
Forest plot of MDI at 12 months of age with risk of bias subgroup analysis

**Fig. 5:**
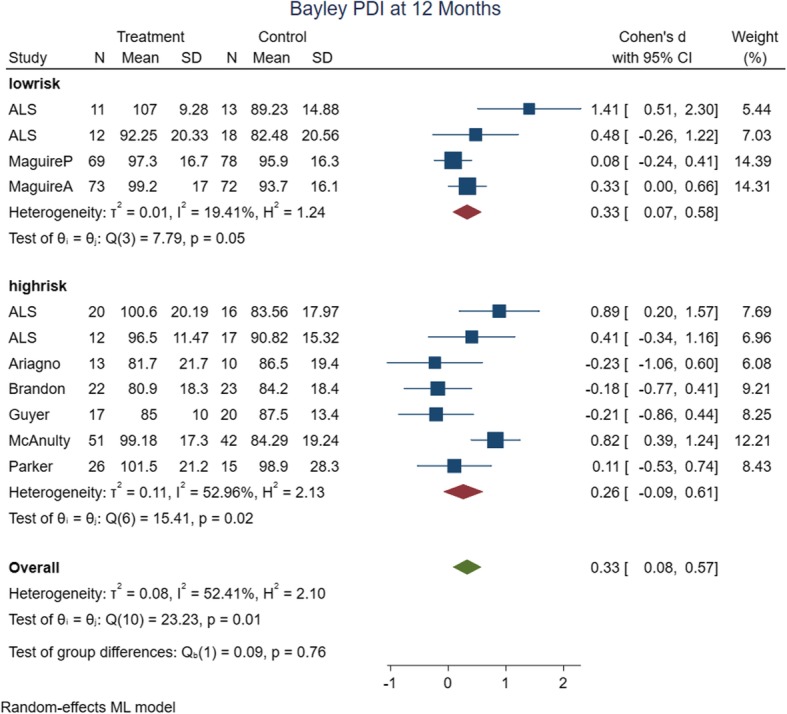
Forest plot of PDI at 12 months of age with risk of bias subgroup analysis

**Fig. 6:**
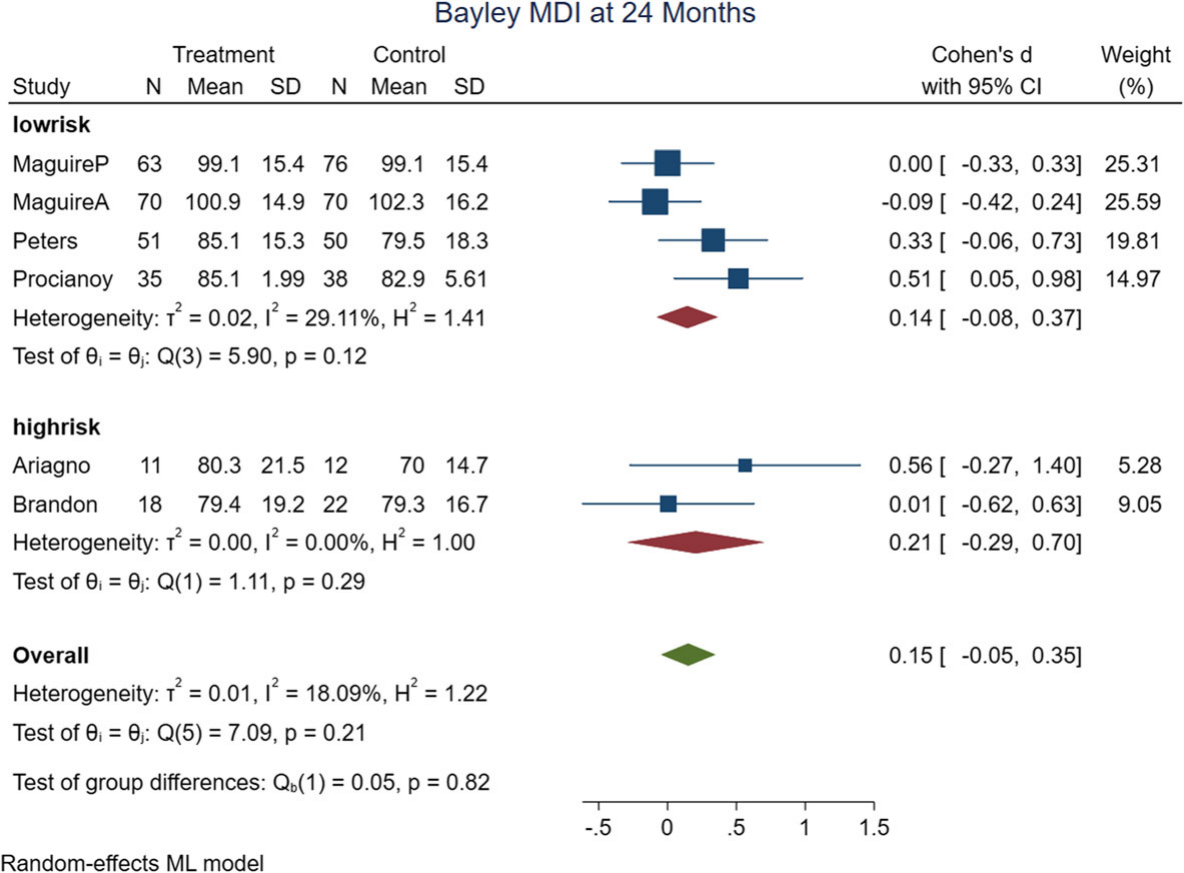
Forest plot of MDI at 24 months of age with risk of bias subgroup analysis

**Fig. 7:**
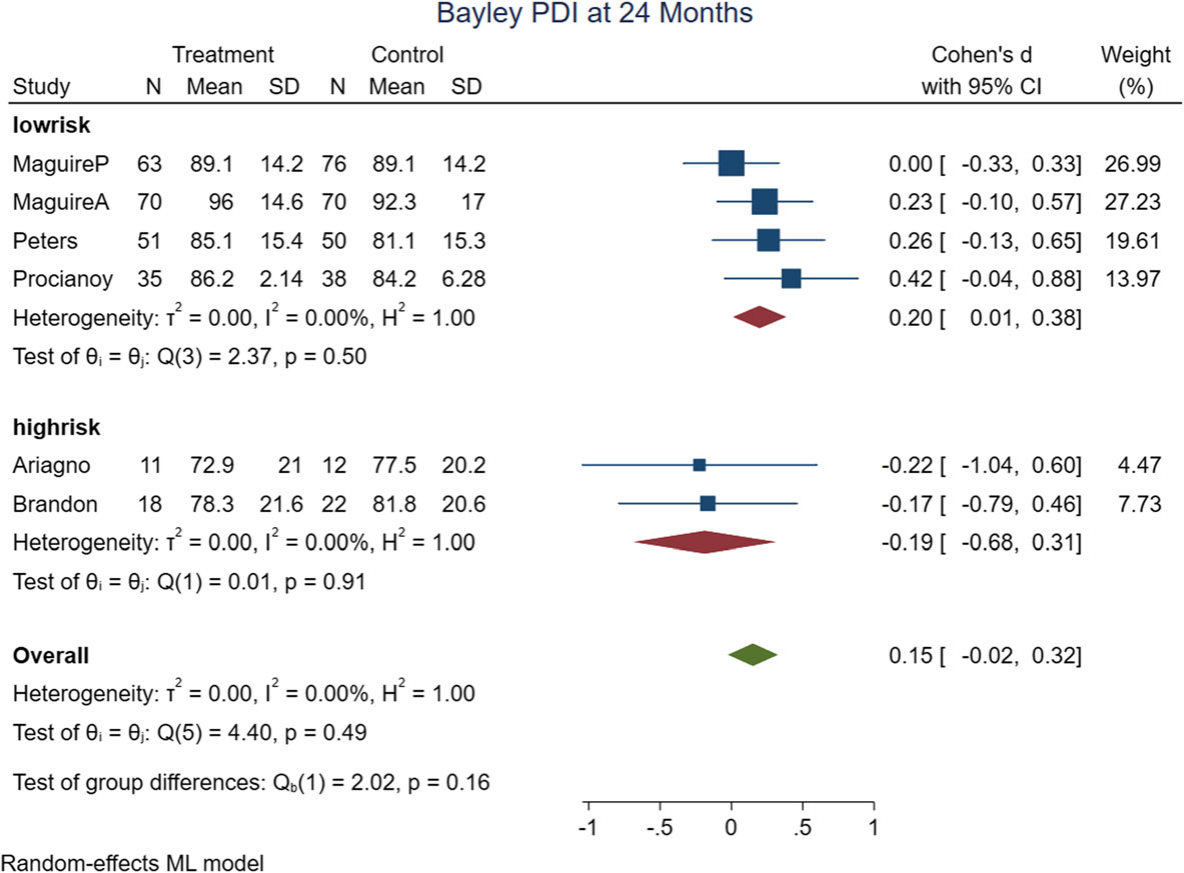
Forest plot of PDI at 24 months of age with risk of bias subgroup analysis

The meta-analysis’ primary and subgroup analyses findings are summarized in Table 2. In assessing the causes of heterogeneity for MDI at 12 months of age, variables such as risk of bias, intervention date and type, method of delivery, birth weight, gestational age, and assessment time were evaluated by subgroup analyses. Only subgroup analyses with the date of intervention (studies ≤2000), and assessment time (9 months); decreased the heterogeneity of MDI at 12 months of age. The rest of the outcomes had low heterogeneity in their primary analysis. The forest plots of the risk of bias subgroup analyses are shown in Figs. 4, 5, 6 and 7.

**Table 2:** Meta-analysis main results and sub-group analyses

Analysis Type	Number of trials	SMD (95% CI)	*P* value	I square
MDI on 12 months of age (intervention versus control)				
Primary analysis	11	0.55 (0.23 to 0.87)	0.001^a^	71.61%
Subgroup analysis by risk of bias				
low risk studies	4	0.62 (− 0.07 to 1.30)	1 = 0.06^b^	1 = 86.72%
high risk studies	7	0.56 (0.22 to 0.90)	2 = 0.004^a^	2 = 50.40%
Subgroup analysis by intervention date (year)				
≤ 2000	5	0.91 (0.63 to 1.19)	1 = 0^a^	1 = 0
> 2000	6	0.26 (− 0.10 to 0.63)	2 = 0.161	2 = 65.2%
Subgroup analysis with intervention type				
NIDCAP	7	0.83 (0.33 to 1.32)	1 = 0.001^a^	1 = 77.5%
Environmental	2	−0.03 (− 0.47 to 0.40)	2 = 0.884	2 = 0
Others	2	0.29 (−0.26 to 0.84)	3 = 0.303	3 = 58.5%
Subgroup analysis with method of intervention delivery				
Nurse	2	0.58 (− 0.51 to 1.67)	1 = 0.295	1 = 87%
Nurse/ Mother	7	0.74 (0.28 to 1.20)	2 = 0.002^a^	2 = 74.7%
Environmental	2	- 0.03 (− 0.47 to 0.40)	3 = 0.884	3 = 0
Subgroup analysis by Birth weight				
≤ 1250 g	3	0.68(− 0.06 to 1.42)	1 = 0.071^b^	1 = 78.9
> 1250 g	3	0.74 (− 0.16 to 1.64)	2 = 0.17	2 = 77.6
Subgroup analysis by Gestational age				
≤ 28 w	4	0.64 (0.06 to 1.22)	1 = 0.031*	1 = 71.5%
> 28 w	7	0.49 (0.09 to 0.89)	2 = 0.016^a^	2 = 71.5%
Subgroup analysis by assessment time				
9 m	7	0.85 (0.45 to 1.25)	1 = 0^a^	1 = 60.1%
12 m	4	0.05 (− 0.15 to 0.26)	2 = 0.607	2 = 0
PDI on12 months of age (intervention versus control)				
Primary analysis	11	0.33 (0.08 to 0.57)	0.013*	I = 52.41%
Subgroup analysis by risk of bias				
low risk	4	0.33 (0.07 to 0.58)	1 = 0.039^a^	1 = 19.41%
high risk	7	0.26 (− 0.09 to 0.61)	2 = 0.192	2 = 52.96%
Subgroup analysis by intervention date				
≤ 2000	5	0.47 (0.07 to 0.87)	1 = 0.021^a^	1 = 48.3%
> 2000	6	0.22(−0.10 to 0.55)	2 = 0.178	2 = 56.1%
Subgroup analysis by intervention type				
NIDCAP	7	0.52 (0.14 to 0.90)	1 = 0.007^a^	1 = 62.9%
Environmental	2	−0.19 (−0.63 to 0.24)	2 = 0.384	2 = 0
Others	2	0.29 (− 0.01 to 0.58)	3 = 0.055^b^	3 = 0
Subgroup analysis by method of delivery				
Nurse	2	0.52 (0.01 to 1.04)	1 = 0.047^a^	1 = 50.4%
Nurse/Mother	7	0.41 (0.05 to 0.77)	2 = 0.025^a^	2 = 60.1%
Environmental	2	−0.19(−0.63 to 0.24)	3 = 0.384	3 = 0
Subgroup analysis by Birth weight				
≤ 1250 g	3	0.39(−0.24 to 1.02)	1 = 0.228	1 = 72.6%
> 1250 g	3	0.38 (−0.47 to 1.23)	2 = 0.383	2 = 76.2%
Subgroup analysis by Gestational age				
≤ 28 w	4	0.36(−0.24 to 0.95)	1 = 0.243	1 = 73.8%
> 28 w	7	0.28 (0.01 to 0.55)	2 = 0.043^a^	2 = 40.9%
Subgroup analysis by assessment time				
9 m	7	0.53 (0.15 to 0.90)	1 = 0.006^a^	1 = 56.6%
12 m	4	0.13(−0.10 to 0.35)	2 = 0.276	2 = 9.5%
MDI on 24 months of age (Intervention versus control)				
Primary analysis	6	0.15(−0.05 to 0.35)	0.15	I = 18.09%
Subgroup analysis by risk of bias				
low risk	4	0.14(−0.08 to 0.37)	1 = 0.248	1 = 29.11%
high risk	2	0.21(− 0.29 to 0.70)	2 = 0.431	2 = 0
Subgroup analysis by intervention date				
≤ 2000	2	0.53 (0.12 to 0.93)	1 = 0.011^a^	1 = 0
> 2000	4	0.05 (−0.14 to 0.24)	2 = 0.612	2 = 0
Subgroup analysis by intervention type				
NIDCAP	3	0.19(−0.09 to 0.48)	1 = 0.187	1 = 20.1
Environmental	1	0.01(−0.62 to 0.63)	2 = 0.986	2 = −---
Others	2	0.19(−0.40 to 0.78)	3 = 0.531	3 = 76.6
Subgroup analysis by method of delivery				
Nurse	1	−0.09(− 0.42 to 0.24)	1 = 0.595	1 = −---
Nurse/ Mother	4	0.27 (0.01 to 0.54)	2 = 0.042^a^	2 = 26.7%
Environmental	1	0.01 (−0.62 to 0.63)	3 = 0.986	3 = −---
Subgroup analysis by Birth weight				
≤ 1250 g	3	0.33 (0.06 to 0.60)	1 = 0.016^a^	1 = 0
> 1250 g	3	0(−0.23 to 0.23)	2 = 0.997	2 = 1.6%
Subgroup analysis by Gestational age				
≤ 28 w	3	0.28 (−0.03 to 0.59)	1 = 0.072^a^	1 = 0
> 28 w	3	0.10 (−0.22 to 0.43)	2 = 0.529	2 = 56%
Subgroup analysis by assessment time				
18 m	2	0.24(−0.09 to 0.57)	1 = 0.158	1 = 0
24 m	4	0.15(−0.15 to 0.46)	2 = 0.529	2 = 48.4%
PDI on 24 months of age (Intervention versus Control)				
Primary analysis	6	0.15 (−0.02 to 0.32)	0.089^b^	0
Subgroup analysis by risk of bias				
low risk	4	0.20 (0.01 to 0.38)	1 = 0.037^a^	1 = 0
high risk	2	−0.19(−0.68 to 0.31)	2 = 0.460	2 = 0
Subgroup analysis by intervention date				
≤ 2000	2	0.19(−0.41 to 0.79)	1 = 0.535	1 = 44%
> 2000	4	0.12(−0.07 to 0.32)	2 = 0.203	2 = 0
Subgroup analysis by intervention type				
NIDCAP	3	0.08(−0.16 to 0.32)	1 = 0.516	1 = 0
Environmental	1	−0.17(− 0.79 to 0.46)	2 = 0.602	2 = −---
Others	2	0.30 (0.03 to 0.57)	3 = 0.032^a^	3 = 0
Subgroup analysis by method of delivery				
Nurse	1	0.23 (− 0.10 to 0.57)	1 = 0.169	1 = −--
Nurse / Mother	4	0.15(− 0.07 to 0.38)	2 = 0.175	2 = 5.3%
Environmental	1	- 0.17(−0.79 to 0.46)	3 = 0.602	3 = −--
Subgroup analysis by birth weight				
≤ 1250 g	3	0.23(− 0.06 to 0.52)	1 = 0.115	1 = 9.5%
> 1250 g	0	–	2 = −--	2 = −--
Subgroup analysis by Gestational age				
≤ 28 w	3	0.09(−0.22 to 0.40)	1 = 0.571	1 = 0
> 28 w	3	0.18(−0.04 to 0.41)	2 = 0.110	2 = 10.6%
Subgroup analysis by assessment time				
18 m	2	0.12(−0.27 to 0.51)	1 = 0.553	1 = 22.4
24 m	4	0.15(−0.05 to 0.36)	2 = 0.110	2 = 3.3%

Significance level at 0.05^a^

Significance level at 0.1^b^

For publication bias assessment, we used funnel plot and Egger’s test. Funnel plots of analyses are presented in Additional file1: Figure S1. In MDI at 12 months of age, there was publication bias with t = 2.30 and *p* value = 0.047. However, there were no publication bias in PDI at 12 months of age (t = 0.41, *p* value = 0.69), MDI at 24 months of age (t = 1.29, *p* value = 0.267) and PDI at 24 months of age (t = − 0.88, p value = 0.426).

Eight studies data were not in meta-analysis and their results are summarized in Table 1. Abou Turk et al. concluded that MDI was better in intervention group compared with control one in preterm infants at 18–22 months of age however PDI were not different between groups. Brown et al. showed that I preterm infants, MDI and PDI were not different between intervention and control groups at 12 months corrected age. In Fajardo and colleagues study, PDI were better at 12 and 24 months of age. However, MDI had no difference between groups.

Welch et al. used Bayley III to assess the developmental care in NICU and showed that cognitive and language domains were better in intervention group at 18 months corrected age.

### Quality of the evidence with GRADE

The Quality of the evidence table with GRADE is illustrated in Additional file 1: Table S2.

a) 12 months of age quality of the evidence: Eleven studies (*n* = 650 infants) were analyzed in 12 months old. However, only four studies (*n* = 346) had low risk of bias that they were considered for the quality assessments. There is moderate quality of the evidence that MDI at 12 months of age were improved in intervention group (SMD: 0.62, 95% CI: − 0.07 to 1.30, *p* value: 0.06). The reason for downgrading the level of evidence for this outcome was high heterogeneity. In addition, there is high quality of evidence that PDI at 12 months of age were also improved in intervention group (SMD: 0.33, 95% CI: 0.07 to 0.58, *p* value: 0.04).

b) 24 months of age quality of the evidence: six trials were analyzed in 24 months old analysis with 516 participants. Four studies (*n* = 453) had low risk of bias and were considered for the quality assessments. There is high quality of evidence that MDI (SMD: 0.14, 95% CI: − 0.08 to 0.37, *P* value: 0.25) and PDI were better in intervention group (SMD: 0.20, 95% CI: 0.01 to 0.38, P value: 0.04).

## Discussion

The aim of this systematic review was to assess the effects of developmental care in NICU setting on mental and motor development of preterm neonates at 6–12 and 13–24 months of age. The interventions in this review included a wide range of interventions and were also very different from one another, so the results could not be combined for an estimate of overall effectiveness.

To our knowledge, this is the first meta-analysis of NICU developmental care interventions for premature infants with a rigorous search strategy, review methodology, inclusion of Randomized controlled trials and a broad range of developmental care and the unique methods of measurement outcomes. Using GRADE with low risk of bias studies, we evaluated the certainty of the evidence to be high-to-moderate for described outcomes in meta-analysis.

MDI and PDI of preterm infants at 12 months of age were significantly better in developmental care group with moderate effect size. Furthermore, MDI at 24 months of age were better with minimal effect size and no statistically significant result. PDI at 24 months of age were also significantly better with minimal effect size.

The span of this review is 46 years, with tremendous changes in the management and care of preterm neonates in the NICU and changes in survival rate of them. Therefore, we used subgroup analysis by intervention date prior and after 2000 year to decrease the clinical heterogeneity.

Furthermore, developmental interventions were highly variable. Therefore, we performed a subgroup analysis by intervention type to minimize this effect. However because of the small number of studies, we could not have divided them in more similar interventions. In addition, we chose only studies that did the developmental care in NICU setting and excluded studies that implemented the developmental care in NICU and Post NICU setting to compare more similar interventions with regard to duration of the interventions.

In subgroup analysis of MDI at 12 months of age, heterogeneity decreased with intervention date, environmental intervention type, and delivery and assessment time. Furthermore, MDI at 12 months of age were better in subgroup analyses with regard to the difference in effect size, intervention dates prior to 2000 year, NIDCAP intervention type, 9 month assessment time with large effect size, and MDI at 12 months of age were better in subgroup analyses by nurse/mother delivery method, birth weight ≤ 1250 g, gestational age ≤ 28 w, with medium effect size in intervention groups.

In subgroup analyses of PDI at 12 months of age, heterogeneity decreased with environmental and other intervention type, and environmental intervention delivery.

In subgroup analyses of PDI at 12 months of age, with regard to the difference in effect size, PDI of studies with low risk of bias, intervention dates prior to 2000 year, NIDCAP, nurse and nurse/mother delivery method and 9 months assessment time were better with medium effect size in intervention groups. In addition, PDI of studies with gestational age > 28 weeks and other intervention types were better with small effect size in intervention groups.

In intervention type, the NIDCAP at 12 months of age, MDI (SMD 0.83, [CI] 0.39–1.32) and PDI (SMD 0.52, [CI] 0.14–0.90) have large and medium effect size respectively on developmental outcomes, which was different with Symington and Pinelli, who concluded that the neuro-developmental outcome results (up to 12 months corrected age) of the NIDCAP trials were conflicting with respect to benefit [13].

In subgroup analyses of MDI at 24 months of age, with regard to the difference in effect size, intervention dates prior to 2000 year, with medium effect size have difference in intervention group. MDI at 24 months of age were also better in subgroup analyses by Birth weight ≤ 1250 g, gestational age ≤ 28 w with small effect size in intervention groups.

In subgroup analyses of PDI at 24 months of age, PDI were better in low risk and other intervention type’s method with small effect size.

With regard to the magnitude and precision of relation, the intervention date and assessment time may be an important factor in assessing intervention effectiveness. The results of studies that occurred before 2000, at 12 months of age, [MDI (SMD 0.91, [CI] 0.63–1.19) and PDI (SMD 0.47, [CI] 0.07–0.87)]; and at 24 months of age, [MDI (SMD 0.53, [CI] 0.12 to 0.93)] were significantly better. The better outcome may be due to the lower survival rate of very premature and VLBWs, and healthier preterm neonates who survived before 2000; therefore, the developmental care in NICU could have more effect on those healthier preterm neonates compared to more fragile preterm neonates after 2000 [15]. Furthermore, routine cares in NICUs are better in recent years and they obviously differ across studies; therefore, pooling results across periods may result in comparing different groups of infants with respect to outcomes. As more recent studies are published it may be possible to group studies according to their time of perinatal care. Also MDI (SMD 0.85, [CI] 0.45–1.25) and PDI (SMD 0.53, [CI] 0.15–0.90) decreased after 9 months of age. This may be due to the increased effects of social and environmental variables with increasing age on development, or perhaps the effect of intervention is more apparent at 9 months of age. Subgroup analyses at 18 and 24 months of age on MDI and PDI were not different according to the effect size.

Previous systematic reviews that investigated the effects of developmental care during NICU admission [13, 24] concluded that there is limited evidence of the long-term positive effect of NIDCAP on behavior and movement of preterm children at 5 years corrected age, but there is no effect on their cognition. Other individualized developmental care interventions have also demonstrated some effect in enhancing neurodevelopmental outcome [13].

On the other hand, for detection of secondary outcome, we have 9 included trials with a marker of illness severity, such as days in the NICU that have weak magnitude and precision of relation (SMD −.168, [CI] -0.471, 0.0135) and no statistically significant effect (*P* value 0.276) between intervention versus control groups. Consistent with our study, Symington et al. also detected an increase in the length of stay, which were demonstrated in infants receiving developmental care compared to control ones.

For publication bias assessment, Egger’s test showed that there was publication bias in MDI at 12 months of age. The publication bias may be due to small number of studies.

Meta-analysis was limited in this review due to the large variation in interventions and limited number of randomized trials that were included in each intervention category. A limitation with developmental care trials was that it is not possible to mask the recipient of the intervention (in this case the mother and infant) or the person applying the intervention, unless the study includes a comparison group getting another intervention rather than no treatment or by cluster RCTs, that we have no cluster RCTs in this study .

Also, there are limitations to meta-analysis, particularly if there is evidence of publication bias, if the outcomes are too dissimilar, or if the studies are at risk of bias. We have tried to minimize these difficulties by representing that there was little evidence of publication bias, by matching comparable outcomes within narrow age ranges and unique assessment tool, and by evaluating the quality of the studies and highlighting the higher-quality studies.

In this review, BSID were considered for developmental assessment. BSID is a comprehensive developmental measure with high sensitivity to identify or monitor the major improvement of long-term neurodevelopmental outcomes and do not exactly assess minor problems [16]. Use BSID could decrease the heterogeneity of studies, but it may limit the number of studies reviewed.

Our primary outcome was the long-term outcome of developmental care in NICU setting. However, the deficiency of available data above 24 months old limits the capacity to compare results between the studies. Diversity and difficulty in identifying the most effective interventions in this review have been emphasized. It is important to aspects of the programs that are mostly useful, and neonates and families who benefit from such programs, more effectively targeting interventions. Long-term benefits for the child and family should also be considered during the intervention. Future studies may include assessments that evaluate functional outcomes, such as educational, behavioral and social problems.

## Conclusion

In this review, we highlighted that NICU developmental care could have a significant effect on mental and motor development of preterm neonates, especially at 9–12 months of age.

The strengths of this systematic review were a rigorous search strategy, review methodology, inclusion of a broad range of developmental care interventions, and the unique methods of measurement outcomes.

Because of high cost, many low and middle income countries (LMIC) cannot implement post NICU interventions; thus, more research is needed for understanding the actual benefit of the administration of developmental care in NICU setting in LMIC for prevention of developmental morbidity in preterm newborns.

## Supplementary information

**Additional file 1: Table S1**: search strategy in PubMed through 8 October 2017. **Table S2**: Quality of the evidence with GRADE (low risk of bias studies). **Figure S1**: Funnel plot of MDI (a), and PDI (b) at 12 months of ages, MDI (c) and PDI (d) at 24 months of ages. The individual study’s standard error (SE[SMD]) is plotted against the standardized mean difference (SMD) for the study.

## Data Availability

The datasets analyzed during the current study are not public, but are available from the corresponding author on reasonable request.

## Abbreviations

BPD: Broncho-pulmonary dysplasia
BSID: Bayley scales of infant development
DCI: Developmental care interventions
GRADE: Grading quality of evidence and strength of recommendations
LMIC: Low and middle income countries
MDI: Mental developmental index
MESH: Medical subject headings
NICU: Neonatal intensive care unit
NIDCAP: Newborn individualized developmental care and assessment program
PDI: Psychomotor developmental index
PRISMA: Preferred reporting items for systematic reviews and meta-analyses
RCT: Randomized controlled trials
SMD: Standardized mean difference
