# Supplementary material for: Do NICU developmental care improve cognitive and motor outcomes for preterm infants? A systematic review and meta-analysis
Source: BMC Pediatr. 2020 Feb 13;20:67. doi: 10.1186/s12887-020-1953-1 (PMC7017495; doi:10.1186/s12887-020-1953-1)
Supplement: Supplementary file 1 — Additional file 1: Table S1: search strategy in PubMed through 8 October 2017. Table S2: Quality of the evidence with GRADE (low risk of bias studies). Figure S1: Funnel plot of MDI (a), and PDI (b) at 12 months of ages, MDI (c) and PDI (d) at 24 months of ages. The individual study’s standard error (SE[SMD]) is plotted against the standardized mean difference (SMD) for the study. [file 12887_2020_1953_MOESM1_ESM.docx]

**Supplementary files**

**Supplementary Appendix Table 1: search strategy in PubMed through 8 October 2017**

| Search | Query | Items found |
| --- | --- | --- |
| #1 | "Intensive Care Units, Neonatal"[Mesh] OR "neonatal ICU"[tiab] OR "neonatal intensive care units"[tiab] OR "neonatal intensive care unit"[tiab] OR " newborn intensive care units"[tiab] OR "newborn intensive care unit"[tiab] OR "NICU"[tiab] OR "newborn ICU"[tiab] | 21998 |
| #2 | "Intensive Care, Neonatal"[Mesh] OR "neonatal intensive care"[tiab] OR "newborn intensive care"[tiab] | 19227 |
| #3 | "Infant, Premature"[Mesh] OR "Infant, Extremely Premature"[Mesh] OR “Premature Birth"[Mesh] OR "premature"[tiab] OR "preterm"[tiab] OR "neonatal prematurity"[tiab] | 170169 |
| #4 | "Infant, Very Low Birth Weight"[Mesh] OR "Infant, Low Birth Weight"[Mesh] OR "Infant, Extremely Low Birth Weight"[Mesh] OR "low birth weight"[tiab] OR “VLBW”[tiab] OR “LBW”[tiab] | 41673 |
| #5 | 1 OR 2 OR 3 OR 4 | 206118 |
| #6 | "bayley" | 4145 |
| #7 | 5 AND 6 | 1046 |

**Supplementary Appendix Table 2: Quality of the evidence with GRADE (low risk of bias studies)**

| **Certainty assessment** | | | | | | | **№ of patients** | | **Effect** | | **Certainty** | **Importance** |
| --- | --- | --- | --- | --- | --- | --- | --- | --- | --- | --- | --- | --- |
| **№ of studies** | **Study design** | **Risk of bias** | **Inconsistency** | **Indirectness** | **Imprecision** | **Other considerations** | **developmental care** | **routine care in NICU** | **Relative (95% CI)** | **Absolute (95% CI)** |  |  |
| **MDI at 12 months of age (low risk of bias studies) (assessed with: BSID)** | | | | | | | | | | | | |
| 4 | randomised trials | not serious | serious ^a^ | not serious | not serious | none | 165 | 181 | - | SMD **0.58 SD higher** (0.02 lower to 1.19 higher) | ⨁⨁⨁◯ MODERATE |  |
| **PDI at 12 months of age (low risk of bias studies) (assessed with: BSID)** | | | | | | | | | | | | |
| 4 | randomised trials | not serious | not serious | not serious | not serious | none | 165 | 181 | - | SMD **0.42 SD higher** (0.02 higher to 0.81 higher) | ⨁⨁⨁⨁ HIGH |  |
| **MDI at 24 months of age (low risk of bias studies) (assessed with: BSID)** | | | | | | | | | | | | |
| 4 | randomised trials | not serious | not serious | not serious | not serious | none | 219 | 234 | - | SMD **0.16 SD higher** (0.11 lower to 0.42 higher) | ⨁⨁⨁⨁ HIGH |  |
| **PDI at 24 months of age (low risk of bias studies) (assessed with: BSID)** | | | | | | | | | | | | |
| 4 | randomised trials | not serious | not serious | not serious | not serious | none | 219 | 234 | - | SMD **0.2 SD higher** (0.01 higher to 0.38 higher) | ⨁⨁⨁⨁ HIGH |  |

**CI:** Confidence interval; **SMD:** Standardised mean difference

**Explanations**

a. The heterogeneity is high because I square is above 75%.

a MDI (12 months)


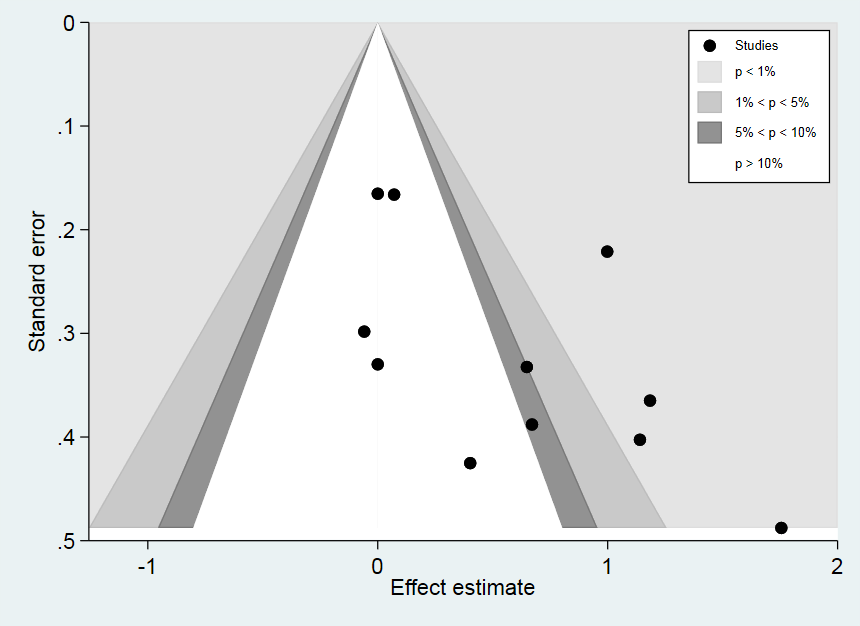


b PDI (12 months)


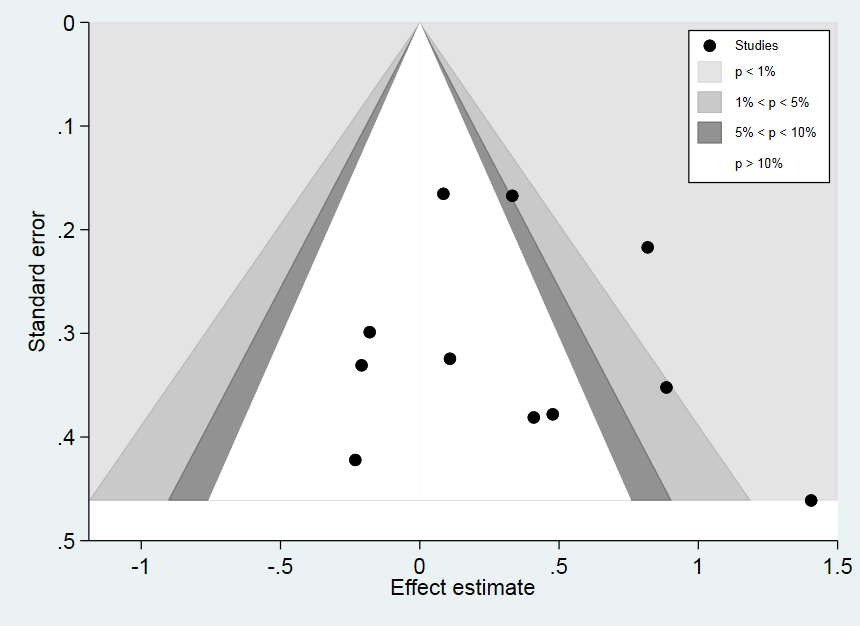


c MDI (24 months)


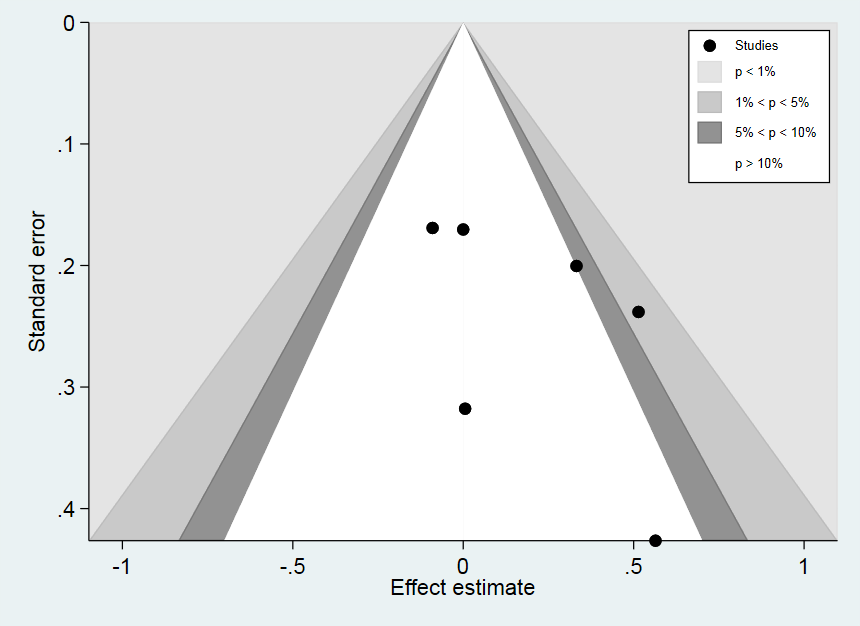


d PDI (24 months)


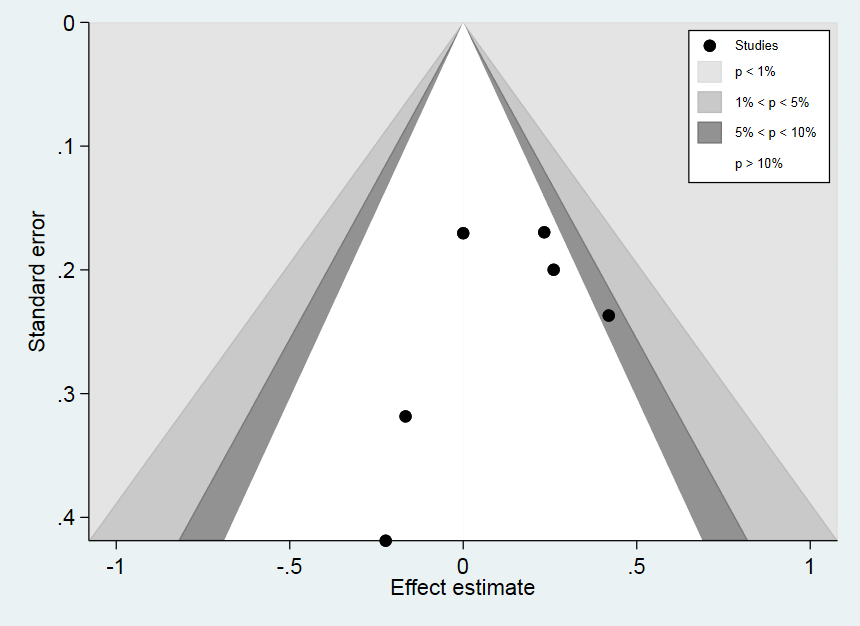


Supplementary Figure 1: Funnel plot of MDI (a), and PDI (b) at 12 months of ages, MDI (c) and PDI (d) at 24 months of ages. The individual study's standard error (SE[SMD]) is plotted against the standardized mean difference (SMD) for the study.
